# A novel nomogram on predicting extrahepatic metastasis in colorectal cancer with liver metastasis for selective application of ^18^F-FDG PET/CT

**DOI:** 10.7150/ijms.104453

**Published:** 2025-01-01

**Authors:** Rongtao Zhu, Yipu Zhao, Jie Zhao, Libo Wang, Sidong Wei, Bin Zhang

**Affiliations:** 1 Department of Hepatobiliary and Pancreatic Surgery, The First Affiliated Hospital of Zhengzhou University, Zhengzhou, Henan 450052, P.R. China.; 2 Department of General Surgery, Henan Provincial Cancer Hospital, Henan 450052, P.R. China.; 3 Department of Hepatobiliary and Pancreatic Surgery, Henan Provincial People's Hospital, Zhengzhou, Henan 450052, P.R. China.

**Keywords:** ^18^F-FDG PET/CT, colorectal cancer liver metastasis, extrahepatic metastases, Nomogram

## Abstract

**Background:** A more accurate assessment of extrahepatic metastases (EHMs) with colorectal cancer liver metastases (CRLMs) improve patient prognosis without unnecessary surgery and economic burden. At present, PET-CT can only be used as a second-line modality. We aimed to construct a predictive model for EHMs, and provide guidance for the selective application of ^18^F-FDG PET/CT.

**Methods:** The clinical data of patients with CRLMs between December 2018 and February 2023 were retrospectively retrieved from the medical records of three large-capacity hospitals. Moreover, we explored the need for ^18^F-FDG PET/CT to be used selectively for detecting EHMs with CRLMs.

**Results:** A total of 471 patients from two hospitals were included in the training set, 174 of whom had CRLMs and EHMs. Notably, the percentages of patients with positive serum CEA, CA19-9 and CA-125 levels were significantly greater in patients with CRLMs and EHMs than in those with liver-limited metastases. Univariate and multivariate logistic regression analyses revealed that the serum levels of CEA and CA-125 and multiple liver metastases were independent risk factors for EHMs. Additionally, we recruited 190 patients with CRLMs from one of the hospitals as the validation set. The nomogram model achieved stable and accurate prediction results in the training and validation sets (AUC = 0.768 and 0.733), and was significantly superior to CEA and CA19-9. Moreover, the sensitivity and specificity of ^18^F-FDG PET/CT for the diagnosis of EHMs were 100% and 88%, respectively.

**Conclusions:** We constructed and validated a nomogram on predicting the risk of EHMs in patients with CRLMs, which can guide clinicians to selective application of ^18^F-FDG PET/CT.

## Introduction

Approximately 50% of patients with colorectal cancer (CRC) develop liver metastases (CRLMs) at some point during their disease 1,2. Surgery is the main curative option for treating patients with CRLMs 3,4. Appropriately selected patients with resectable hepatic metastases and limited extrahepatic disease can benefit from aggressive surgical resection in combination with chemotherapy, which can lead to improved survival 5,6. In other patients, metastases are considered unresectable and neoadjuvant chemotherapy is the main treatment. Therefore, the challenge is defining which patients are the best candidates for each approach 2. Further accurate assessments of the preoperative metastatic patterns of CRC can be performed to develop more precise and individualized treatments and improve patient prognosis without unnecessary surgery.

Studies have shown that positron emission tomography-computed tomography (PET-CT) is useful for the clinical guidance and treatment of CRLMs 7. However, there exists controversy, and PET-CT scans at this point can only be used as a second-line modality. Stepwise imaging is the recommended policy in terms of therapeutic possibilities, rather than using all imaging modalities for all patients 8,9. Therefore, PET-CT should be used selectively in patients with CRLMs. Moreover, a significant economic burden has been placed on healthcare systems and populations due to the cost of treating CRLMs 10. It is necessary that the specific indications of PET/CT for CRLMs need to be further explored for precision preoperative assessment and to avoid unnecessary medical resources. To date, markers of the risk of EHM, including clinical characteristics and histopathological and molecular parameters, are important indicators for the appropriate use of PET-CT examinations and the selection of effective treatments 11,12. Elevated carcinoembryonic antigen (CEA) levels indicate poor prognosis and the presence of recurrent disease 12,13. However, the predictive value of CEA, carbohydrate antigen 19-9 (CA19-9), carbohydrate antigen 125(CA-125) and alpha fetoprotein (AFP) levels for EHMs in patients with CRLMs is unclear.

In this study, we retrospectively analyzed the clinical data of CRLM patients in three large-capacity hospitals and explored the pattern of distant metastasis. The independent risk factors of EHMs were obtained by univariate and multivariate logistic regression analysis, and then a novel nomogram model predicting the risk of EHMs in CRLM patients was subsequently constructed and validated. This nomogram is a reliable predictive tool and shows more accurate predictive performance than the currently widely used CEA and CA19-9 11-13. Furthermore, we validated the sensitivity and specificity of ^18^F-FDG PET/CT examination for the diagnosis of EHMs with CRLMs. Through the nomogram tool, clinicians can determine the risk of EHMs in patients with CRLMs, and provide additional assessments such as PET/CT for appropriate patient selection.

## Materials and methods

### Patients

The clinical data of patients with CRLM who were diagnosed and treated at the First Affiliated Hospital of Zhengzhou University, Henan Provincial People's Hospital and Henan Cancer Hospital from December 2018 to February 2023 were retrospectively analyzed. According to different hospital, patients were divided into training and validation groups. The complete clinical data of the patients were collected and the metastatic patterns of cancer in patients with colon and rectal cancer were analyzed by ^18^F-FDG PET/CT. The imaging data of the patients were evaluated independently by two experienced radiologists. This study was approved by the ethics committee of the First Affiliated Hospital of Zhengzhou University (No. 2021-KY-013).

### Inclusion and exclusion criteria

The following data were collected: (1) histopathological examination and pathological diagnosis of colon adenocarcinoma or rectal adenocarcinoma; (2) imaging diagnosis of colon cancer or rectal cancer with liver metastases; (3) measurement of the levels of the tumor markers CEA, CA19-9, CA-125 and AFP as well as CT or MRI examination to evaluate the primary tumor or postoperative recurrence and; (4) complete general clinical data available for the patient. The exclusion criteria were as follows: (1) diagnosis of CRC combined with another primary malignant tumor; (2) patients without imaging for primary cancer; (3) patients without CT or MRI imaging evaluation for liver and EHMs.

### Observation indices

Data were collected on the following indexes: (1) general characteristics such as age, sex, comorbidities; (2) clinical and lymphatic metastasis by imaging CT or MRI; (3) the cancer differentiation grade of the tumor; (4) the number and size of liver metastases and EHMs, as revealed by imaging studies; (5) serum levels of tumor markers such as CEA, CA19-9, CA-125 and AFP; (6) ^18^F-FDG PET/CT results. ^18^F-FDG PET/CT results were obtained from a report made by an imaging specialist. Patients were imaged by a 64-slice hybrid PET/CT scanner (Biograph, TruePoint64, Inc. Germany) following the standard protocol 14.

### Statistical analysis

SPSS version 23.0 statistical software (SPSS Inc., Chicago, IL, USA) was used for the statistical analysis. The chi-square test was used to analyze categorical variables. Respectively, the cut-off values of serum CEA, CA19-9, CA-125 and AFP were 5ng/mL, 37 U/mL, 35 U/mL and 20 ng/mL respectively, according to the manufacturer's instructions and previous studies 15,16. Univariate and multivariate logistic regressions were used to estimate odds ratios (ORs) and 95% confidence intervals (CIs) for the associations between EHMs and other potential risk factors. Receiver operating characteristic (ROC) curves were generated and the areas under the curves (AUCs) were calculated to investigate the efficiency of each independent risk factor and our model for the prediction of EHMs in patients with CRLMs. The nomogram, C-index, calibration curves and ROC curves were calculated and visualized with R 4.0.2 software. All tests were two sided, and P < 0.05 was considered statistically significant.

## Results

### Patient collection

**Figure 1:**
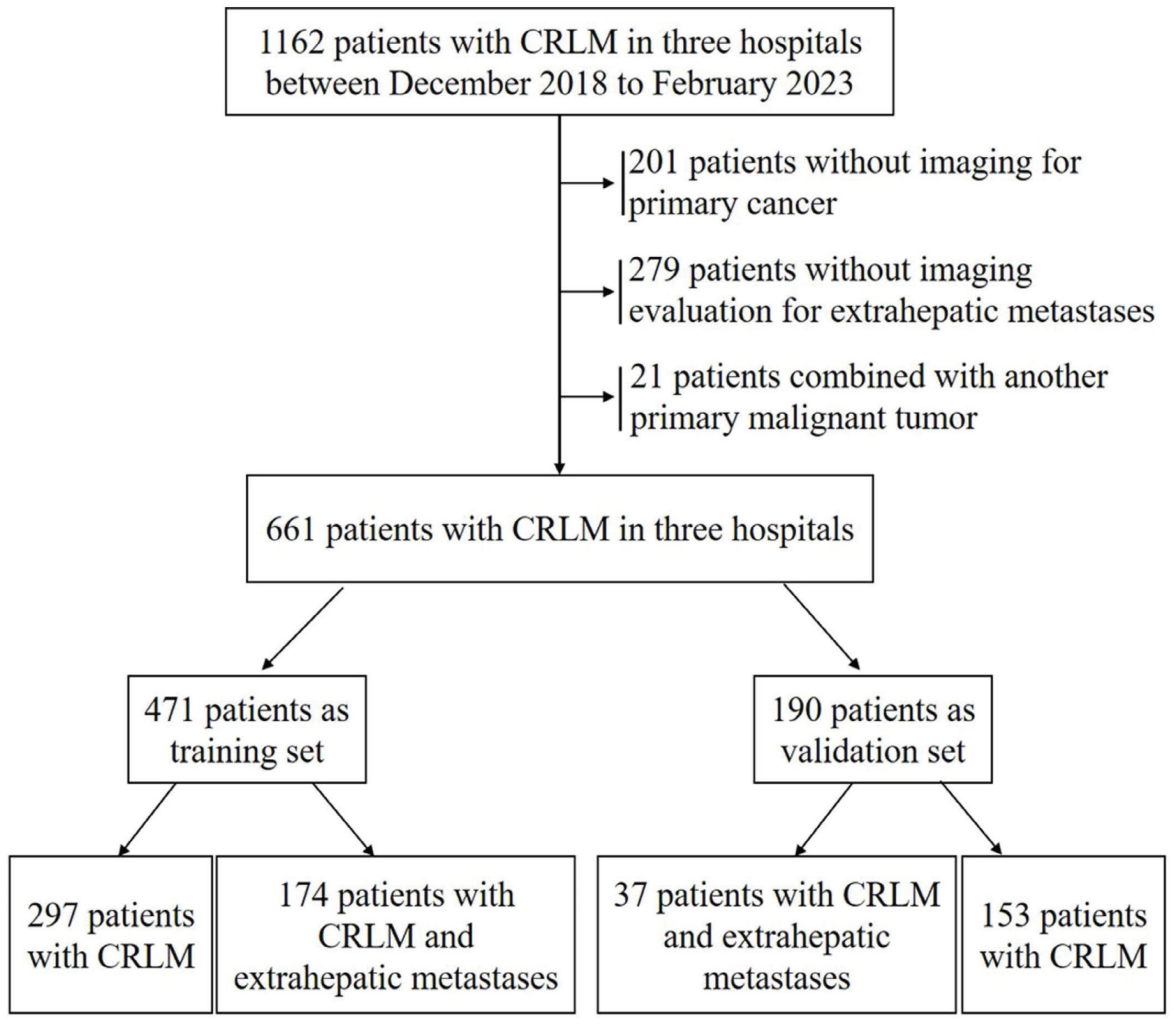
Patient flowchart for the study and subgroup analysis.

A total of 1162 patients were enrolled from three large-capacity hospitals in Henan Province, China, from December 2018 to February 2023. In all, 501 patients who failed to meet the inclusion criteria were excluded. After excluding patients, the present analysis involved 661 patients. Among these, 471 patients were divided into a training set and 190 patients were divided into a validation set (Figure 1).

### Patient demographic and clinical data

A total of 471 CRLM patients from the First Affiliated Hospital of Zhengzhou University and Henan Cancer Hospital were included in the training set, including 273 cases with rectal cancer and 198 cases with colon cancer; 283 patients were male, and 188 patients were female. Among these patients, the median age was 60 years old, ranging from 20 to 87 years. There were no significant differences in the general clinical data between colon and rectal cancer patients. In addition, we additionally collected data from 190 colorectal cancer cases from Henan Provincial People's Hospital as the validation cohort, and the analysis revealed that their baseline data were not significantly different from those of the training cohort (P > 0.05) (Table 1).

### Patterns of extrahepatic metastases of CRLMs

Among the 471 cases with CRLMs in training set, there were 174 cases with EHMs. The lungs were the most common metastatic target organ in patients with EHMs, accounting for 70.7% and 63.4% of EHMs in patients with rectal cancer and colon cancer, respectively (P = 0.31). There was no significant difference in the proportion of metastases in the skeletal system and other organs between the two groups (P > 0.05). Notably, compared with patients with rectal cancer, patients with colon cancer were more likely to have extrahepatic peritoneum metastases (34.1% vs 19.6%, P = 0.03). Overall, 19 patients in both patient groups developed distant metastases more than three target organs with EHMs. However, the differences between the rectal cancer groups and the colon cancer groups were not detected (P = 0.611) (Table 2).

### Levels of serum CEA, CA19-9, CA-125 and AFP in patients with CRLMs

We divided the training set into positive and negative groups according to the cutoff value of each tumor marker, and further calculated the positive rates of each marker in the liver-limited metastasis and EHMs groups. The results showed that the positive rates of serum CEA, CA19-9 and CA-125 in patients with CRLMs with EHMs were significantly higher than those in patients with CRLMs with liver-limited metastases (87.9% vs. 66.3%, 71.8% vs. 47.8%, 58.6% vs. 25.3%; P < 0.05). However, the positive rate of serum AFP did not differ significantly between the two groups of patients (21.3% vs. 16.8%, P = 0.284; Table 3). These results indicate that CRLMs patients with positive serum CEA, CA19-9 and CA-125 levels are more likely to develop EHMs than those with normal negative marker levels. Moreover, an analysis of the differences of these serum markers between the training set and the validation set revealed that there were no detectable differences (P > 0.05, Table 3).

### Risk factors for extrahepatic metastases in patients with CRLMs

To investigate the potential risk factors for EHMs in patients with CRLMs, univariate and multivariable logistic regression analyses were performed using potentially significant variables. These univariate analyses revealed that age, pathological stage and serum AFP levels were not significantly correlated with the occurrence of EHMs in patients with CRLMs (P > 0.05). Furthermore, statistically significant gender, CEA, CA19-9, CA-125, multiple liver metastases and lymphatic metastases in univariate analysis were included in multivariate analysis, and the results showed that CEA, CA-125 and multiple liver metastases were independent risk factors for EHMs in patients with CRLMs, with ORs of 3.41 (95% CI 1.89-6.36), 4.12 (95% CI 2.67-6.42) and 4.53 (95% CI 1.98-12.32), respectively (Table 4).

### Construction and validation of the nomograms

**Figure 2:**
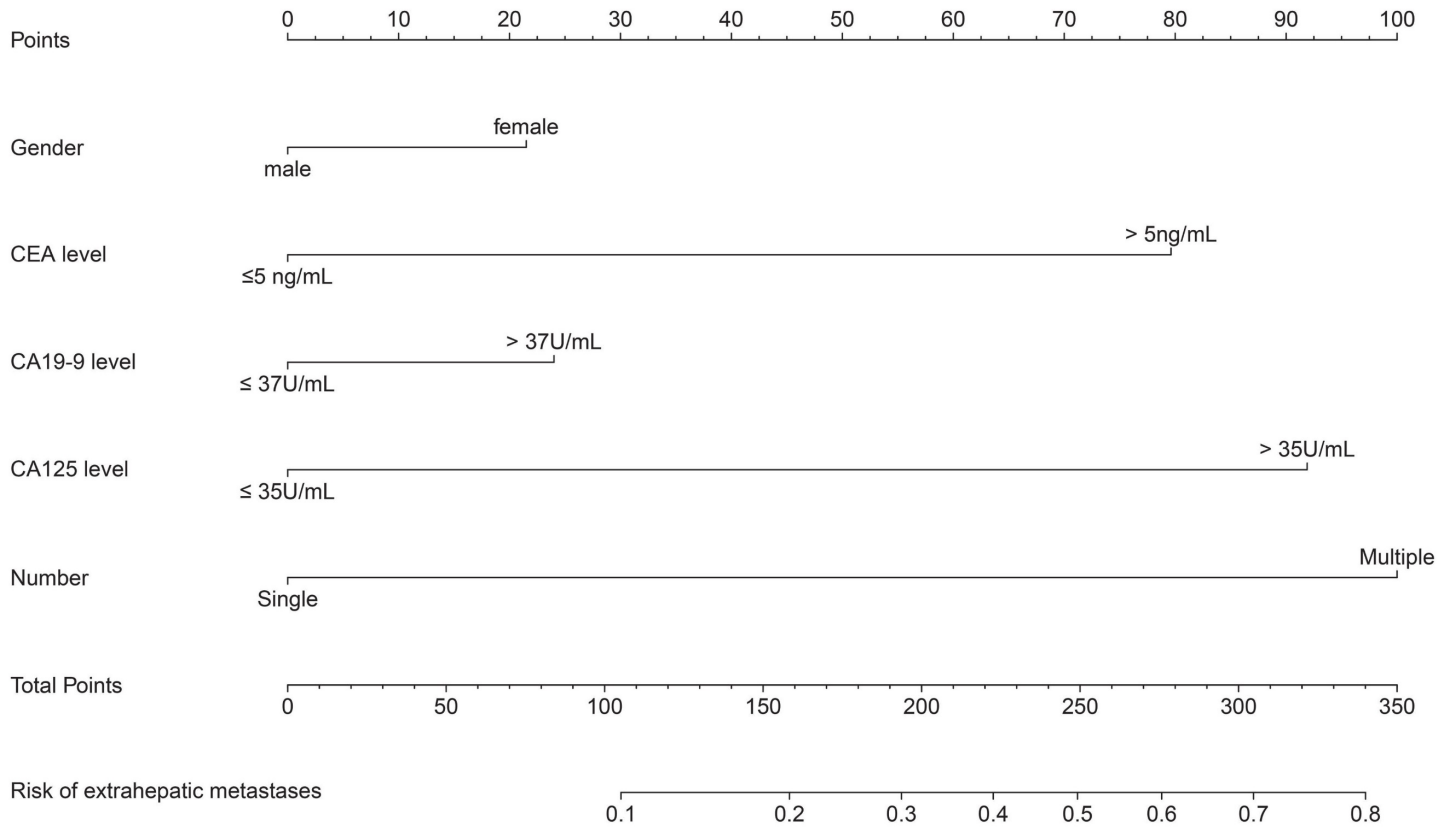
Nomogram for predicting the risk of extrahepatic metastasis in patients with colorectal cancer liver metastasis.

Three independent risk factors in multivariate logistic, CEA, CA-125 and multiple liver metastases. It is worth noting that although gender and CA19-9 did not reach statistical significance, there was a consistent trend (P=0.127 and 0.164, respectively). Considering the widely recognized clinical significance of gender and CA19-9, in the process of constructing our nomogram model to predict EHMs in patients with CRLMs, in addition to including serum CEA, CA-125 levels, and multiple liver metastases as independent risk factors, we also included gender and serum CA19-9 simultaneously (Figure 2). The model showed excellent prediction accuracy, which C-index was 0.768 (95% CI: 0.724-0.811, P < 0.001) for the training set with a slope of 1.000000e+00 and intercept of -3.907113e-12, and C-index was 0.733 (95% CI: 0.651-0.814, P < 0.001) for the validation set with a slope of 1.000000e+00 and intercept of -1.443098e-15.

We next generated ROC curves for each individual factor and nomogram model and calculated the AUCs to evaluate the diagnostic value of each factor for the diagnosis of EHMs in patients with CRLMs. As shown in Table 5, gender, CEA, CA19-9, CA-125, and multiple liver metastases had AUCs of 0.443, 0.608, 0.620, 0.667, and 0.579, respectively, when predicting EHMs in CRLMs patients alone, which were much lower than the 0.768 in our nomogram model.

**Figure 3:**
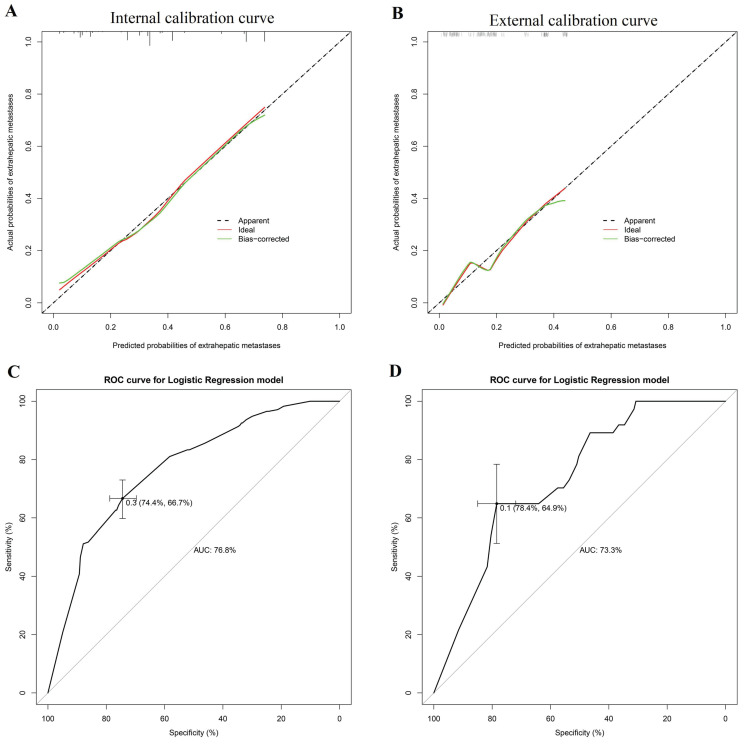
Calibration curves and ROC curves of nomogram in training and validation datasets. The internal calibration curve (A) in training set and external calibration curve (B) in validation dataset. The internal ROC curve (C) in training set and external ROC curve (D) in validation dataset.

The calibration curves of both the training and validation datasets reflected that the nomogram model could predict the EHM of CRLMs patients well (Figure 3 A-B). Moreover, we conducted internal validation including 10-fold Cross-Validation, leave-one-out cross-validation and bootstrap validation on our training set, and took the average AUC and the average C-index of more than 200 iterations as the evaluation criteria. According to 10-fold cross validation and bootstrap validation with iterations times 200, the average AUC were 0.755 (95% CI 0.745-0.765) and 0.770 (95%CI 0.767-0.773), the average C-index were 0.721 (95% CI 0.711-0.736) and 0.735 (95% CI 0.732-0.738). Leave-one-out cross-validation, iterations times 471, the resulting AUC was 0.720 and the obtained C-index was 0.720. Therefore, according to the average AUC and C-index were greater than 0.7 in the three validation methods.

Furthermore, analysis of the validation set indicted that our nomogram model also showed a more robust predictive power in the validation set (AUC = 0.733, Figure 3 C-D). Overall, the above results indicate that our nomogram model can more accurately predict the risk of EHMs in patients with CRLMs and is superior to biomarkers such as CEA and CA19-9, which are currently used alone in clinical practice.

### Application of ^18^F-FDG PET/CT for the diagnosis of EHMs

**Figure 4:**
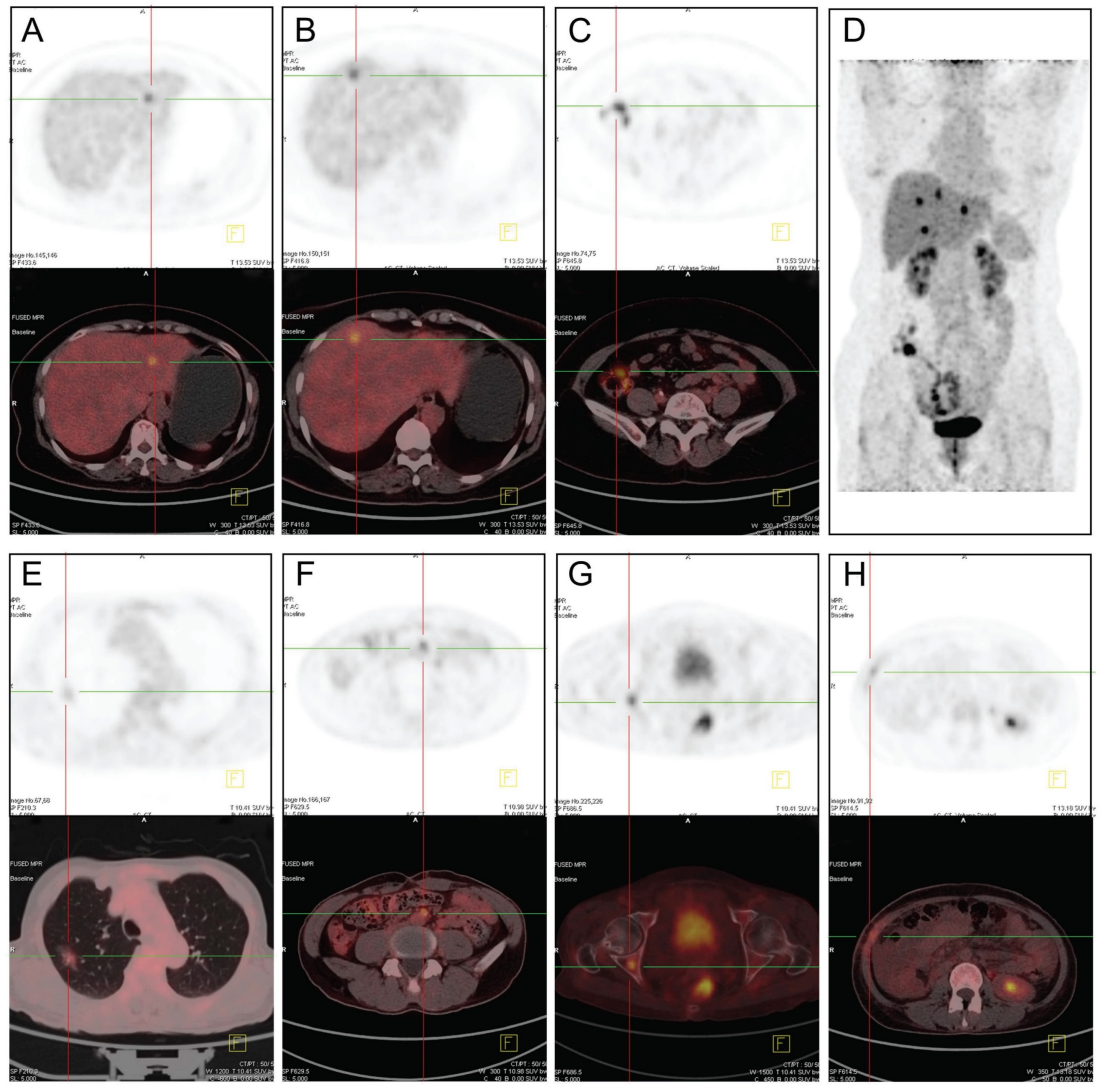
^18^F-FDG PET-CT images of patients with CRLMs and EHMs. (A-D) Different metastatic sites from the same patient. A, SUV_max_ =7.9. B, SUV_max_ =8.5. C, colon cancer with SUV_max_ =9.6. (E) Lung metastases, SUV_max_ = 5.6. (F) Retroperitoneal lymph nodes metastases, SUV_max_= 6.7. (G) Bone metastases on the right acetabulum, SUV_max_= 6.5. (H) Peritoneal and abdominal wall metastases, SUV_max_= 4.9. SUVmax: maximum standardized uptake value.

Among the 471 patients with CRLMs, 60 patients underwent ^18^F-FDG PET/CT examination, including 35 cases with EHMs and 25 cases with liver-limited metastases. ^18^F-FDG PET/CT was able to detect the metabolic activity of CRLMs and EHMs. The most frequent site of metastasis was lung in patients with EHMs. And peritoneum, distant lymph nodes and skeletal system were common metastasis lesions in some patients. A few patients developed several metastases in multiple organs at the same time (Figure 4). All 35 cases with EHMs were diagnosed correctly with ^18^F-FDG PET/CT. Unfortunately, of the 25 cases with liver-limited metastases, 3 cases were misdiagnosed; these patients were confirmed to have inflammatory disease after anti-infection drug treatment. The sensitivity and specificity of ^18^F-FDG PET/CT for the diagnosis of EHMs of CRLMs were 100% and 88%, respectively (Table 6).

## Discussion

There is a role for treatment in select patients with EHMs, although the expectations should be different than those for patients with liver-limited metastases 2,3. Radiological imaging plays an important role in the determination of liver disease and in identifying the presence of extrahepatic disease that would preclude curative resection 8. PET/CT examination using ^18^F-fluorodeoxyglucose (^18^F-FDG) has been used extensively for preoperative evaluations of colorectal cancer 17. ^18^F-FDG PET/CT has obvious advantages in the diagnosis of liver and EHMs in CRC, and it is highly accurate for the detection of liver metastases on an individual patient basis 7,18,19. However, PET/CT is should not be routinely used for the preoperative evaluation of CRLMs 9. A previous meta-analysis revealed that PET/CT scan was not found to improve the overall survival rate, and that open-close surgeries were not significantly reduced 20. Therefore, the specific indications for PET/CT in patients with CRLMs need to be explored in order to avoid unnecessary medical resources and improve the preoperative assessment. Moreover, whether widely used biomarkers such as CEA, CA19-9, CA-125 and AFP in digestive system tumors can provide more information about the spread of EHMs in patients with CRLMs remains unclear. In this study, we explored the potential clinical utility of CEA, CA19-9, CA-125 and AFP and certain key clinical features as predictive biomarkers for EHM in patients with CRLMs for precision preoperative assessment and selection of ^18^F-FDG PET/CT examination for treatment.

Many previous studies have confirmed that approximately 50% of colorectal cancers developed liver metastases, with lung metastases being the most common site of EHMs, followed by the peritoneal, pelvic, and skeletal systems 1,2,14,21. Our results verified that the lung was the most common site of EHMs, and lung metastasis was found in 63.4% and 70.7% of patients with colon and rectal cancer with liver metastasis, respectively. In addition, patients with colon cancer were more prone to peritoneal metastasis (34.1% vs. 19.6%). These findings are consistent with previous findings indicating that patients with liver metastases from colon cancer are more likely to have peritoneum metastases than those with rectal cancer.

Recently, several studies have been conducted to evaluate the role of common tumor markers CEA, CA19-9, CA-125 and AFP in the diagnosis, prognosis and postoperative recurrence detection of patients with CRLMs 11-13,15,16. However, most studies emphasize the relationship between serum maker levels and liver metastasis, and very few studies focus on the relationship between serum marker levels and the risk of EHMs, most of these studies are derived from public databases or single-center small sample studies. Considering the great challenge posed by evaluating whether patients with CRLMs have distant metastases poses for individualized clinical treatment, our findings have significant clinical implications. In this study, based on clinical data from three large-capacity hospitals, we observed that CRLMs patients with EHMs had significantly higher positive rates of CEA, CA19-9, CA-125 and AFP than patients with localized liver metastasis. Further univariate and multivariate logistic regression analysis revealed that serum CEA, CA-125 levels and the clinical features of multiple liver metastases were independent risk factors for concurrent extrahepatic metastasis in patients with CRLMs. We then included the above independent risk factors, along with gender and CA19-9, which did not reach statistical significance but had a consistent trend and were clinically significant, to construct the nomogram model. Fortunately, the consistency of evaluation indicators, such as the calibration curve, C-index and AUC, shows that our nomogram model can stably and accurately predict the risk of EHMs in patients with CRLMs in both the training and validation datasets, and is significantly superior to biomarkers such as CEA and CA19-9, which are currently used alone in clinical practice. In clinical practice, based on our nomogram model, we can intervene in advance and predict the risk of EHMs in patients with CRLMs. Generally speaking, in clinical practice, for patients with a total score greater than 250 points and a risk of extrahepatic metastasis exceeding 50%, we recommend that patients undergo ^18^F-FDG PET/CT examination to develop further diagnosis and treatment strategies based on the treatment willingness of the patients and their families.

After screening cases at the greatest risk for EHMs, we explored the application of ^18^F-FDG PET/CT for the diagnosis of EHMs in patients with CLRMs. In total, 60 patients in our study were examined by ^18^F-FDG PET/CT. Two cases of pulmonary inflammatory lesions were misdiagnosed as CRLM with lung metastasis due to inflammation-induced metabolic activity. Because of PET/CT with 18F-FDG reflecting biological activity of tumors, and FDG uptake is increased in inflammatory tissue, which increased the risk of false positive findings 17,22. Our findings regarding the usefulness of ^18^F-FDG PET/CT were in agreement with the findings of a previous study 7,23,24. In contrast, other studies found that among patients with potentially resectable CRLMs, the use of ^18^F-FDG PET/CT compared with CT alone did not result in frequent changes in surgical management 25. Furthermore, with the limitations of the decreased sensitivity of PET/CT for the detection of small colonic lesions <10 mm in diameter, preoperative chemotherapy sometimes affects the accuracy of PET/CT 26. Therefore, personalized medicine requires the evaluation of patients with CRLMs using our nomogram model, in which patients at greater risk of EHMs benefit from ^18^F-FDG PET/CT examination, rather than the indiscriminate application of ^18^F-FDG PET/CT to all cases with CRLMs.

This retrospective study was conducted to analyze the clinical characteristics of CRLMs and to provide precise staging for the preoperative diagnosis of patients. Patients without liver metastases were excluded, limiting the metastatic patterns of CRC. In addition, this framework solely pertained to the diagnosis of EHMs in patients with CLRMs. Moreover, the confirmation of a pathological diagnosis in some patients was clearly difficult to obtain, the metastasis of some cases depended on imaging diagnosis without pathological basis. The relationships among different metastatic patterns, the related risk factors for tumor metastasis and the prognosis of the patients still need to be further explored.

## Conclusion

In summary, distant metastases in patients with CRLMs have unique clinical characteristics, for whom the lung is the most common metastatic site. Elevated serum levels of CEA, CA-125 and multiple liver metastases were found to be independent risk factors for EHMs. Our nomogram model can stably and accurately predict the risk of EHMs in patients with CRLMs and is superior to biomarkers such as CEA and CA19-9, which are currently used alone in clinical practice. Therefore, we strongly recommend the use of our nomogram model to evaluate patients with CRLMs and to perform ^18^F-FDG PET/CT examinations in patients at high risk of EHMs to guide the individualized treatment of patients with CRLMs.

## Figures and Tables

**Table 1:** Demographic and clinical data of patients

Characteristics	Training set (n)				Validation set (n)			P (total)
	CRLM	CRLM + EHM	P		CRLM	CRLM + EHM	P	
Gender			0.014				0.018	0.216
Male	191	92			106	18		
Female	106	82			47	19		
Age (years, range)	60(20-87)	60(29-86)	0.876		61(27-87)	60(45-83)	0.935	0.25
Comorbidities			0.29				0.292	0.936
Hypertension	48	33			26	7		
Diabetes	30	19			16	4		
Cardiopulmonary disease	16	10			7	3		
Anemia	6	5			3	2		
Primary cancer			0.087				0.261	0.378
Colon cancer	116	82			67	20		
Rectal cancer	181	92			86	17		
Multiple liver metastases			<0.001				0.003	0.381
Single	119	41			55	4		
Multiple	178	133			98	33		
Lymphatic metastasis			0.036				0.033	0.42
No	110	48			62	8		
Yes	187	126			91	29		
Tumor differentiation			0.739				0.392	0.437
Poor	166	100			79	22		
Others	131	74			74	15		

**Table 2:** Extrahepatic metastasis patterns in patients with CRLM

Location of EHM	Colon cancer(n)	Rectal cancer(n)	X-squared	P
Total	82	92	--	--
Lung	52	65	1.031	0.31
Peritoneum	28	18	4.74	0.03
Skeletal system	5	7	0.154	0.695
Others	8	13	0.782	0.377
≥3 organs	10	9	0.259	0.611

**Table 3:** Serum levels of CEA, CA19-9, CA-125 and AFP in patients

	Training set (n)				Validation set (n)			P (total)
	CRLM	CRLM + EHM	P		CRLM	CRLM + EHM	P	
CEA (ng/ml)			<0.001				0.006	0.76
≤5	100	21			47	4		
>5	197	153			106	33		
CA199 (U/ml)			<0.001				0.019	0.249
≤37	155	49			65	8		
>37	142	125			88	29		
CA125 (U/ml)			<0.001				<0.001	0.761
≤35	222	72			107	14		
>35	75	102			46	23		
AFP (ng/ml)			0.232				0.193	0.096
≤20	247	137			119	25		
>20	50	37			34	12		

CEA: carcinoembryonic antigen; CA19-9: carbohydrate antigen 19-9; CA-125: carbohydrate antigen 125; AFP: alpha fetoprotein

**Table 4:** Univariate and multivariate logistic regression analyses of risk factors for EHM in training set patients.

Variable	Univariate				Multivariate		
	OR	95%CI	P		OR	95%CI	P
Gender	1.61	1.10- 2.35	0.015		0.72	0.47-1.10	0.127
Age	1.00	0.99-1.02	0.753		--	--	--
Cancer differentiation grade (poor vs other)	1.09	0.64-1.88	0.757		--	--	--
CEA	3.70	2.25-6.33	0.000		3.41	1.89-6.36	0.000
CA19-9	2.78	1.87-4.18	0.000		1.40	0.87-2.27	0.164
CA-125	4.19	2.82-6.28	0.000		4.12	2.67-6.42	0.000
AFP	0.23	0.83-2.14	0.233		--	--	--
Multiple liver metastases	6.65	3.03-17.56	0.000		4.53	1.98-12.32	0.001
Lymphatic metastases	2.37	1.26- 4.66	0.009		0.81	0.51-1.29	0.380

**Table 5:** The AUCs of biomarkers and nomogram for predicting the risk of EHM in patients with CRLM

	AUC	Standard error	95% CI	P
Gender	0.443	0.024	0.397-0.489	0.015
CEA	0.608	0.018	0.572-0.644	0.000
CA19-9	0.620	0.022	0.576-0.664	0.000
CA-125	0.667	0.023	0.623-0.711	0.000
Multiple liver metastases	0.579	0.013	0.553-0.605	0.000
Nomogram model	0.768	0.022	0.724-0.811	0.000
External cohort	0.733	0.041	0.651-0.814	0.000

AUC: area under curve; CRLM: colorectal liver metastases; CEA: carcinoembryonic antigen; CA19-9: carbohydrate antigen 19-9; CA-125: carbohydrate antigen 125

**Table 6:** Application of ^18^F-FDG PET/CT for the diagnosis of liver and EHM of CRLM

	EHM (n)	Liver-limited metastasis (n)
PET/CT positive	35	3
PET/CT negative	0	22

^18^F-FDG PET/CT, ^18^F-fluorodeoxyglucose positron emission tomography/computed tomography; X-squared = 48.632, p-value <0.001
